# AGO1 may influence the prognosis of hepatocellular carcinoma through TGF-β pathway

**DOI:** 10.1038/s41419-018-0338-y

**Published:** 2018-02-27

**Authors:** Miao Wang, Lyu Zhang, Zeyang Liu, Jiamin Zhou, Qi Pan, Jia Fan, Rongyu Zang, Lu Wang

**Affiliations:** 1 Department of Liver Surgery, Shanghai Cancer Center, Shanghai Medical College, Fudan University, 270 Dongan Road, Shanghai, China; 2 Research Center for Translational Medicine, Shanghai East Hospital, Tongji University School of Medicine, Shanghai, China; 3 Liver Cancer Institute, Zhongshan Hospital, Fudan University, 180 Fenglin Road, Shanghai, China; 4 Division of Gynecologic Oncology, Department of Obstetrics and Gynecology, Zhongshan Hospital, Fudan University, 180 Fenglin Road, Shanghai, China

## Abstract

AGO1 is a major component of RNA-induced silencing complexes and plays a crucial role in solid tumors. The aim of our study was to investigate AGO1 functions in hepatocellular carcinoma (HCC). Using small interfering RNA, AGO1 functions were investigated in HCCLM3 cell lines. Cell proliferation, immigration, and invasion significantly decreased after AGO1 depletion using MTT, wound-healing, and transwell assay. The associated proteins in the epithelial–mesenchymal transition (EMT) and the activation of its signal pathways were measured using western blot. After AGO1 depleted, increased E-cadherin and decreased N-cadherin, Vimentin, Snail, and Zeb1 were founded. In its upstream pathway, the phosphorylation of ERK1/2(Thr202/Tyr204), Smad2(S425/250/255), and Smad4 were significantly inhibited. Meanwhile, inhibitor of ERK1/2(LY3214996) significantly inhibited the growth and migration of the AGO1 cells. The nuclear importing of Smad4 was blocked and furthermore, the transcription of Snail was also influenced for the decrease of combination between Smad4 and the promotor region of Snail. After Snail was overexpressed, the invasion of HCCLM3 cells was significantly rescued. Immunohistochemistry in tissue microarrays consisting of 200 HCC patients was used to analyze the associations between AGO1 expression and prognosis. Intratumoral AGO1 expression was an independent risk factor for overall survival (*P* = 0.008) and recurrence-free survival (*P* < 0.001). In conclusion, AGO1 may promote HCC metastasis through TGF-β pathway, and AGO1 may be a reliable prognostic factor in HCC.

## Introduction

Liver cancer is the second leading cause of cancer death worldwide and in less-developed countries and the sixth leading cause of cancer death in more-developed countries among men. During 2012, an estimated 782 500 new liver cancer cases and 745 500 deaths occurred worldwide, with China alone accounting for about 50% of the total number of cases and deaths. Most (70–90%) primary liver cancers occurring worldwide are hepatocellular carcinoma (HCC)^1,2^. The high postsurgical recurrence rate is a major problem, thus HCC patients undergoing curative resection have a poor outcome. Meanwhile, the survival benefit of several postsurgical biotherapies and targeting therapies has been certified currently^3^. Consequently, the identification of biomarkers that can predict the outcome and effectiveness of adjuvant therapies is crucial for preventing postsurgical recurrence and increasing the life span of patients with HCC.

MicroRNAs (miRNAs) modulated cellular gene expression through the RNA interference (RNAi) pathway and are important for carcinogenesis and tumor progression^4,5^. Although repressing miRNAs may be a novel cancer therapy, it is difficult to inhibit multiple miRNAs, which could be a more effective way to against cancer, in cancer cells simultaneously because each miRNA may independently contribute to tumor development^6^. The AGO family (AGO1, AGO2, AGO3, and AGO4) is the essential protein component of the RNAi machinery, and it can regulate the biogenesis of multiple miRNAs. AGO family members are present in all RNA-induced silencing complexes (RISCs)^7^ and are required for the production of mature miRNAs^8^. AGO1 is a major component of RISCs and is a well-studied AGO in mammals. The relationship between AGO1 and cancer has been reported in several recent investigations. Li et al.^9^ have found that AGO1 may be a novel early diagnostic marker because it is significantly associated with colon cancer tissue occurrence. AGO1 also plays a crucial role in lung and breast cancers^10,11^. However, AGO1 has just been reported to overexpress in intratumoral tissues and there are limited data on the role of AGO1 in HCC^12^. Therefore, exploring the relationship between AGO1 and HCC to identify an effective biomarker may be more promising for treatment compared to suppressing miRNAs.

## Materials and methods

### Cell lines and chemicals

HCC cell line with high metastatic potentials, HCCLM3, was used, which was established by the Liver Cancer Institute of Zhongshan Hospital (Shanghai, China)^13–15^. The cells were cultured in Dulbecco’s modified Eagles medium, supplemented with 10% fetal bovine serum in a humidified atmosphere of 5% CO_2_ at 37 °C. LY3214996 and SB203580 were purchased from Calbiochem (La Jolla, CA, USA).

### Patients, specimens, and follow-up

From January 2000 to November 2001, 200 consecutive patients undergoing curative resection with pathologically confirmed HCC were enrolled in this study. The paired intratumoral and peritumoral tissues were collected from the patients. Patients did not receive presurgical anticancer treatment. The clinicopathological features were described in our previous study^16^. In our hospital, the lower limit of quantification 5 × 10^2^ IU/ml was used to define hepatitis B virus (HBV) replication. All patients were recruited into the current study after signing the informed consent. This study was approved by the Research Ethics Committee of Zhongshan Hospital. All patients were followed until March 2008 with a median follow-up time of 59.1 months (range, 1–91 months). The follow-up procedures were previously described^16^.

### siRNA-mediated silencing of AGO1 and AGO2

The target siRNA sequences are listed in Table 1. RNA duplexes were designed and synthesized by the Genepharma Company (Shanghai, China). Transfection of siRNA into HCCLM3 cells was performed using Lipofectamine 2000 (Invitrogen, Carlsbad, CA) according to the manufacturer’s instructions.

**Table 1:** Sequences of AGO1-targeting siRNA

siRNA	Sequence
AGO1 siRNA-1 sense	5ʹ-CCCAGAUACUCCACUAUGATT-3ʹ
AGO1 siRNA-1 antisense	5ʹ-UCAUAGUGGAGUAUCUGGGTT-3ʹ
AGO1 siRNA-2 sense	5ʹ-CCCACCCAUUUGAGUUUGATT-3ʹ
AGO1 siRNA-2 antisense	5ʹ-UCAAACUCAAAUGGGUGGGTT-3ʹ
AGO2 siRNA-1	5ʹ-UCGAAGUAUUCCGCGUACGUG-3ʹ
AGO2 siRNA-2	5ʹ-CGUACGCGGAAUACUUC GAAA-3ʹ
Control sense	5ʹ-UUCUCCGAACGUGUCACGUTT-3ʹ
Control antisense	5ʹ-ACGUGACACGUUCGGAGAATT-3ʹ

### Western blot analysis

For western blot analysis, the protein was collected from HCCLM3 cells transfected with different AGO1-targeting siRNAs for 24 h. The primary antibodies used were AGO1, AGO2, E-cadherin, N-cadherin, Vimentin, Twist, Snail, p-ERK1/2(Thr202/Tyr204), ERK1/2, p-P38, P38, p-Smad2(S425/250/255), p-Smad2(Thr 8), p-Smad2(T220/T179), Smad2, p-Smad4, Smad4, GAPDH, Tubulin, and LaminA/C (Cell Signaling Technology, Danvers, MA; 1:1000).

### Cell proliferation, migration, and invasion

The MTT assay was used for cell proliferation analysis^17^. Absorbance was measured at 490 nm at the designated time points (24, 48, 72, and 96 h). Transwell assays were used for cell invasion analysis^16^. The wound-healing assay was used to assess the migration of cells. The cells were seeded in six-well plates with a density of 2 × 10^5^ cells/well and grown to confluency. Then pipette tips of 200 µl (Corning, USA) were used to scrape over the adherent cells to make a wound. Migrating cells at the wound front were recorded at 0 and 24 h after wounding. Migrating ratio was calculated as followed: ([average width of linear wound at 0 h − average width of linear wound at 24 h]/average width of linear wound at 0 h).

### Chromatin immunoprecipitation and quantitative real-time polymerase chain reaction

Immunoprecipitation of viral DNA was performed by chemically crosslinking DNA–protein complexes formed in cells using 1% formaldehyde. The cells were incubated for 10 min at 37 °C and then washed once in phosphate-buffered saline containing protease inhibitors (Thermo Scientific). Cells were re-suspended in SDS lysis buffer (50 mM Tris-HCl [pH 8.1], 1% SDS, and 10 mM EDTA) and sonicated four times, 10 s each, using Sonifier 450 apparatus (Branson). Cell lysates were cleared by centrifugation and the collected supernatants were diluted 10-fold in chromatin immunoprecipitation (ChIP) dilution buffer (16.7 mM Tris-HCl [pH 8.1], 0.01% SDS, 1.1% Triton X-100, 167 mM NaCl, and 1.2 mM EDTA). Smad4-associated DNA was immunoprecipitated using a rabbit polyclonal antibody generated against the full-length protein. The immune complexes were collected on protein G agarose beads (Millipore). The Snail promotor region binding to Smad4 was assessed by quantitative real-time polymerase chain reaction (qRT-PCR). The primers are listed as follows:

5ʹ-GTTTCCCTCGTCAATGCCACGCTCTC-3ʹ;

5ʹ-GCAGCAGCGCCGCCAACTCCCTTAA-3ʹ.

### Immunohistochemistry

Four hundred pairs of representative formalin-fixed and paraffin-embedded intratumoral and peritumoral tissue cores, isolated from areas adjacent to tumors, were constructed into tissue microarrays. Duplicate cylinders from two different areas were obtained. The immunohistochemistry (IHC) and photographing protocols were described previously^16^. The primary antibody used for IHC was AGO1 (1:500). The AGO1 was determined as previously described^16^.

### Statistical analysis

Values were expressed as the mean ± standard error of the mean. Unpaired *t*-test was used to compare quantitative variables. Pearson *χ*^2^-test or Fisher exact test was applied to compare qualitative variables. The patients’ survival curve was plotted using the Kaplan–Meier method, and the log-rank test was used to determine the significant difference among groups. The Cox regression model was used to perform multivariate analysis. Analysis was performed using SPSS 16.0 for Windows (SPSS Inc., Chicago, IL). *P* < 0.05 was considered statistically significant.

## Results

### AGO1 depletion reduced cell proliferation, migration, and invasion

**Fig. 1:**
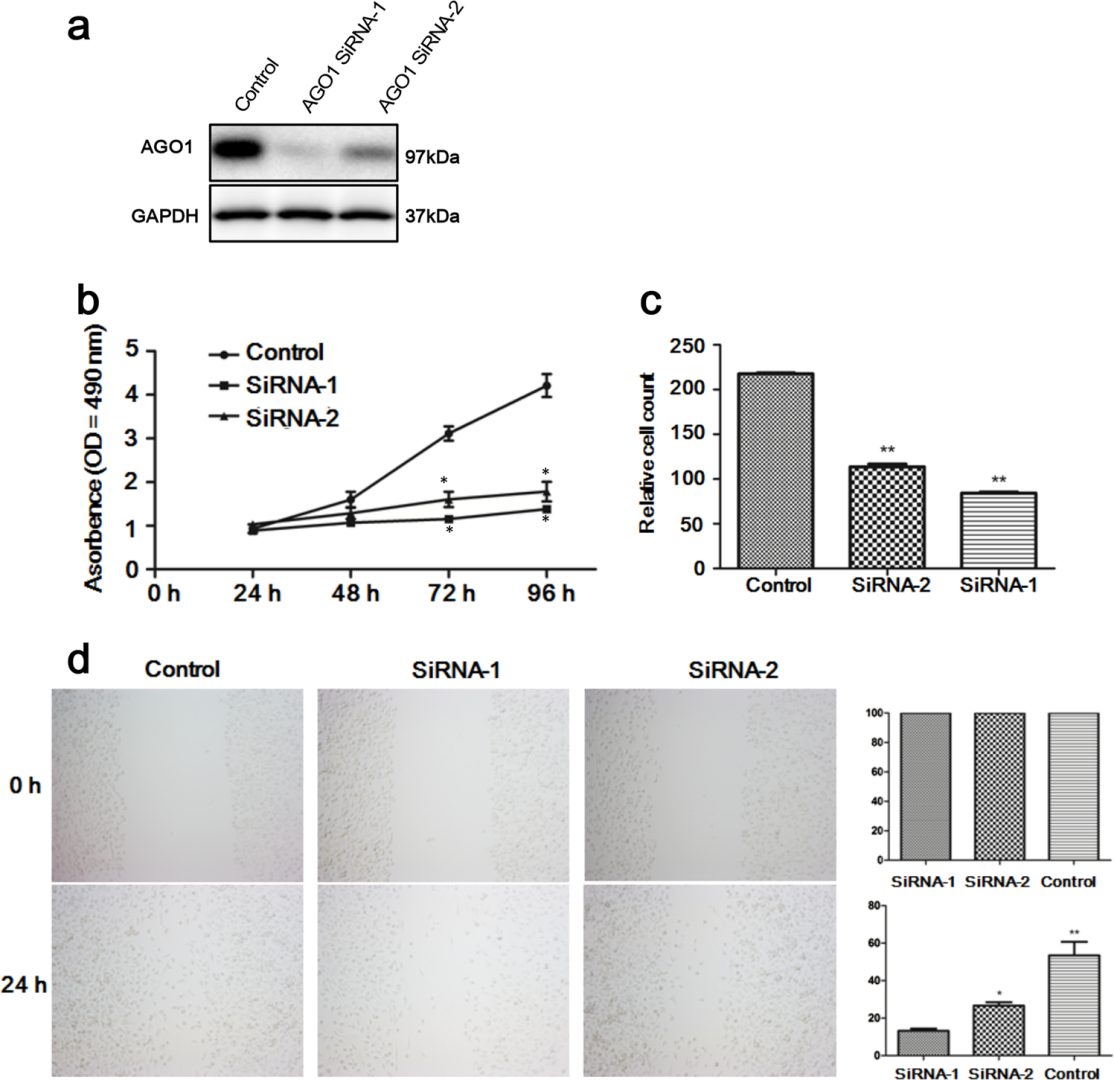
The cell functions of HCCLM3 after AGO1 siRNA transfection. **a** AGO1 expression was inhibited significantly after siRNA transfection. **b** Cell proliferation was measured using the MTT assay. **c** Cell invasion was measured using Matrigel transwell invasion assays. **d** Cell migration was measured using wound-healing assay. *P < 0.05; **P < 0.001

To investigate the functions of AGO1 in HCC, we depleted AGO1 in HCCLM3 cells using siRNA. The suppressive effects of the siRNAs were validated at 24 h by western blot. AGO1 siRNA-1 and AGO1 siRNA-2 demonstrated significant suppression effects compared with control siRNA sequences and AGO1 expression was significantly lower after AGO1 siRNA-1 transfection than after AGO1 siRNA-2 transfection (Fig. 1a). Downregulation of AGO1 caused a significant decrease in cell proliferation at 48 h following transfection (*P* = 0.039; Fig. 1b). Decreased AGO1 expression was accompanied by a reduction in HCC cell invasion, as measured by the transwell assay (*P* < 0.001; Fig. 1c). Similarly, wound-healing assay showed that AGO1 depletion caused a significantly greater distance between the two wound fronts (*P* < 0.001; Fig. 1d). In order to approve the miRNA is responsible for AGO1 is known to be incorporated in RISC. We chose AGO1 KD cells to examine the expression of miR173, miR-981, and miR-317 by RT-PCR assays. Strikingly, depletion of AGO1 markedly decreased the mRNA level of miR173, miR-981, and miR-317 (Supplementary Figure 1).

### AGO1 depletion inhibited the epithelial–mesenchymal transition and phosphorylation of ERK1/2, Smad2, and Smad4

**Fig. 2:**
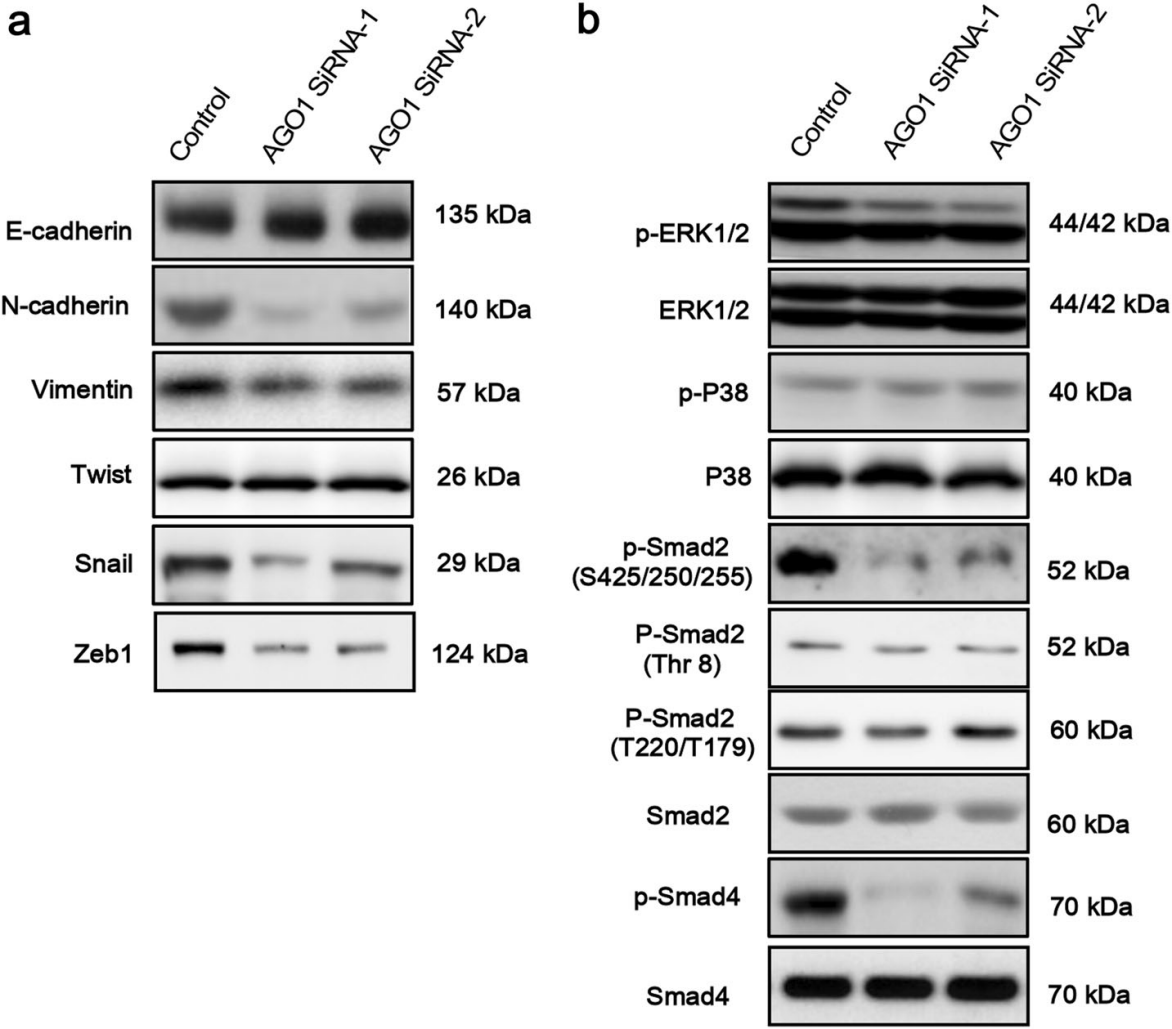
The expression of AGO1, EMT-associated proteins, and TGF-β pathway-associated proteins in HCCLM3 after AGO1 transfected using AGO1 siRNAs as determined by western blot analyses. **a** AGO1 expression was inhibited significantly after siRNA transfection, as well as mesenchymal indicators N-cadherin and Vimentin, and transcription factors Snail and ZEB1, while epithelial indicator E-cadherin increased and another transcription factor Twist did not show any change. **b** The phosphorylation of TGF-β pathway-associated proteins, including ERK1/2, p-Smad2(S425/250/255), and Smad4 was significantly inhibited by AGO1 depletion, and P38, p-Smad2(Thr), and p-Smad2(T220/T179) did not show any change

**Fig. 3:**
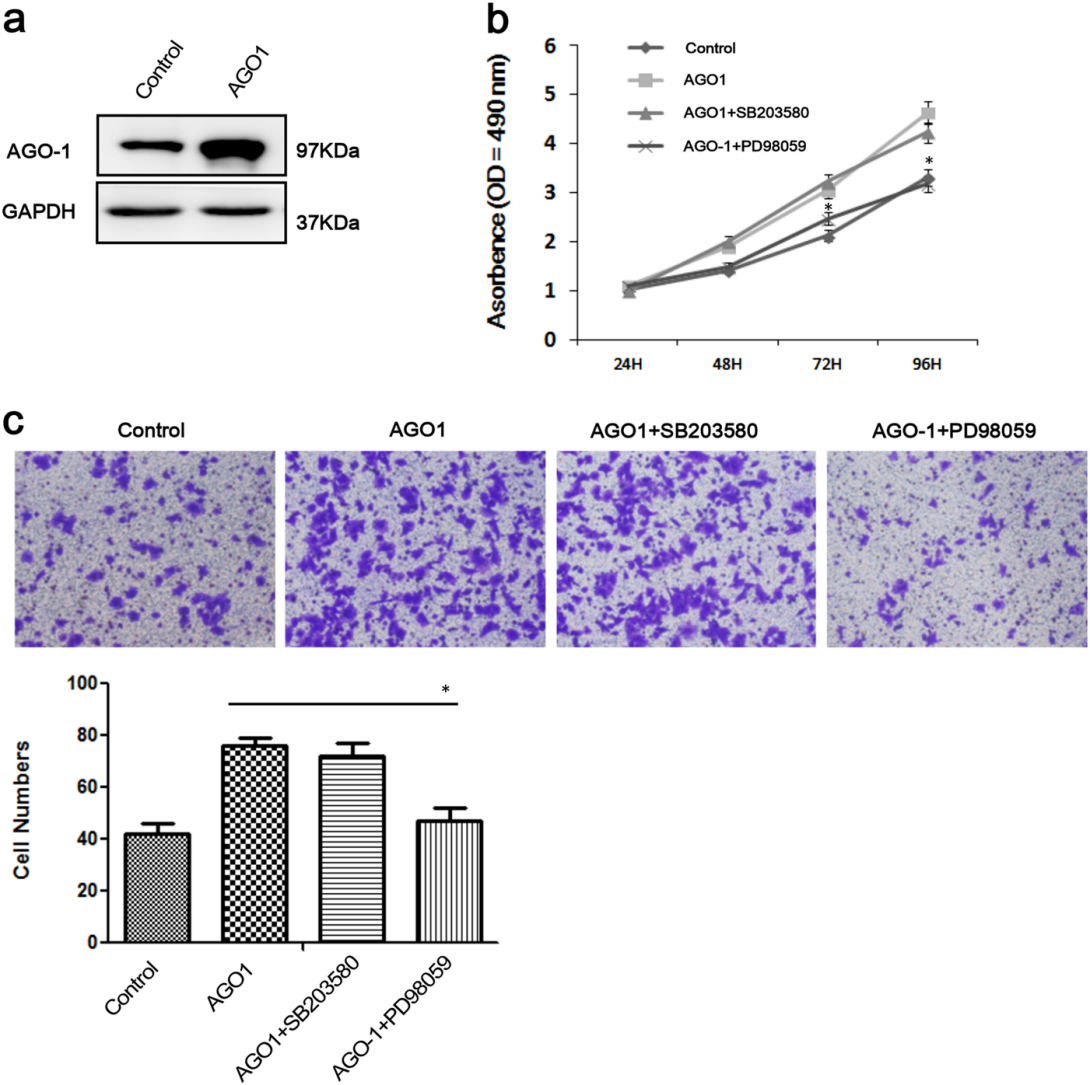
Ago1 promotes cell proliferation and migration though ERK1/2 not P38. **a** Level of AGO1 protein was detected by western blotting assays. **b** LY3214996 (ERK1/2) inhibitor significantly inhibited AGO1 cell growth compared to the normal group, while SB203580 (P38) did not significantly affect growth (*P < 0.05). **c** LY3214996 (ERK1/2) inhibitor significantly inhibited AGO1 cell migration compared to the normal group, while SB203580 (P38) did not significantly affect migration (*P < 0.05)

The alteration of AGO1 expression could influence the functions of HCC tumor cells, including proliferation and invasion. Thus, we further investigated the potential mechanism. By western blot, the expression of epithelial–mesenchymal transition (EMT)-associated proteins altered significantly after AGO1 depletion. Increase of E-cadherin and decrease of N-cadherin and Vimentin were observed. EMT transcription-associated proteins Snail and Zeb1 were also significantly downregulated, while another EMT transcription-associated protein Twist did not show any change (Fig. 2a). Then, we explored the transforming growth factor (TGF)-β pathway-associated proteins, which might alter the expression of transcription factor Snail. The phosphorylation of ERK1/2, other than P38, was dramatically downregulated after AGO1 depletion. Likewise, the phosphorylation of ERK1/2-regulating proteins Smad2(S425/250/255) and Smad4 were also significantly downregulated (Fig. 2b). However, knockdown of AGO1 did not show any effect on phosphorylation of Smad2(T220/T179) and (Thr 8) (Fig. 2b). To further prove that AGO1 regulated Smad2(S425/250/255) via ERK1/2, not P38. We use the inhibitors of LY3214996(ERK1/2) and SB203580(P38) respectively treated with elevated AGO1 cells to assay the cell proliferation and migration. Remarkably, inhibitor of LY3214996(ERK1/2) significantly inhibited the growth and migration of the AGO1 cells when compared with normal group (Fig. 3b, c). However, inhibitor of SB203580(P38) did not significantly impacted the cell proliferation and metastasis of AGO1 cells when compared with normal group (Fig. 3b, c). To prove that this observation is specific for AGO1 not AGO2, we first examined the expression and phosphorylation status of ERK1/2, Smad2, and Smad4 by western blot in AGO2 knockdown cells and in control groups. Interestingly, we found these proteins were not apparently changed (Supplementary Figure 2). These results, taken together, suggest that the EMT pathway and ERK1/2-p-Smad2(S425/250/255)-p-Smad4 have an important role in AGO1 downregulation.

### AGO1 depletion inhibited the nucleus entrance of Smad4 to downregulate the expression of Snail

**Fig. 4:**
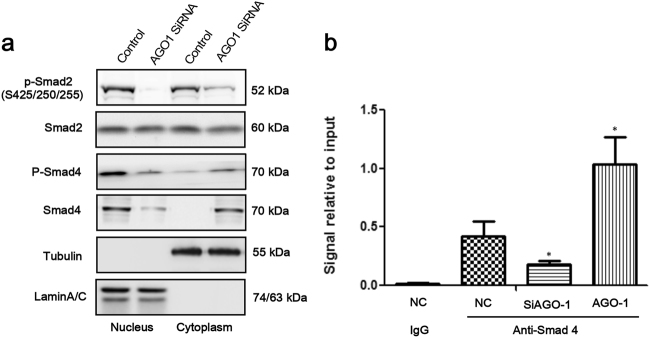
The expression of Smad2/4 in the nucleus and cytoplasm and the result of ChIP. **a** The expression of Smad4 and p-Smad4 was significantly downregulated in the cell nucleus and significantly increased in the cell cytoplasm; Smad2 did not show any alteration; P-Smad2 was significantly downregulated in the both cell nucleus and cytoplasm. **b** After being pulldown by anti-Smad4 antibody and control IgG antibody respectively, the quantity of promoter region of Snail combined to Smad4 was dramatically more that control. *P < 0.05

It is necessary to check whether Smad2 or Smad4 would transfer into the nucleus to play a role in regulating the expression of Snail, which made us extract the cell nucleus and cytoplasm proteins respectively to confirm that. Then, depletion of AGO1 resulted in decreased nuclear Smad4 and p-Smad4, but not Smad2 (Fig. 4a). Correspondingly, the expression of Smad4 in the cell cytoplasm significantly increased (Fig. 4a). Interestingly, p-Smad2 was reduced both in the nucleus and in the cytoplasm (Fig. 4a). ChIP was used to explore whether AGO1 would influence the Smad4 localization to Snail genomic locus. The qRT-PCR results revealed that AGO1 can regulate the level of Smad4 binding Snail, we found that AGO1 overexpression could indeed activate Snail promoter while AGO1 knockdown inactivate Snail promoter (*P* < 0.001; Fig. 4b).

### Overexpressed Snail could reverse the inhibitory effect of AGO1 depletion

**Fig. 5:**
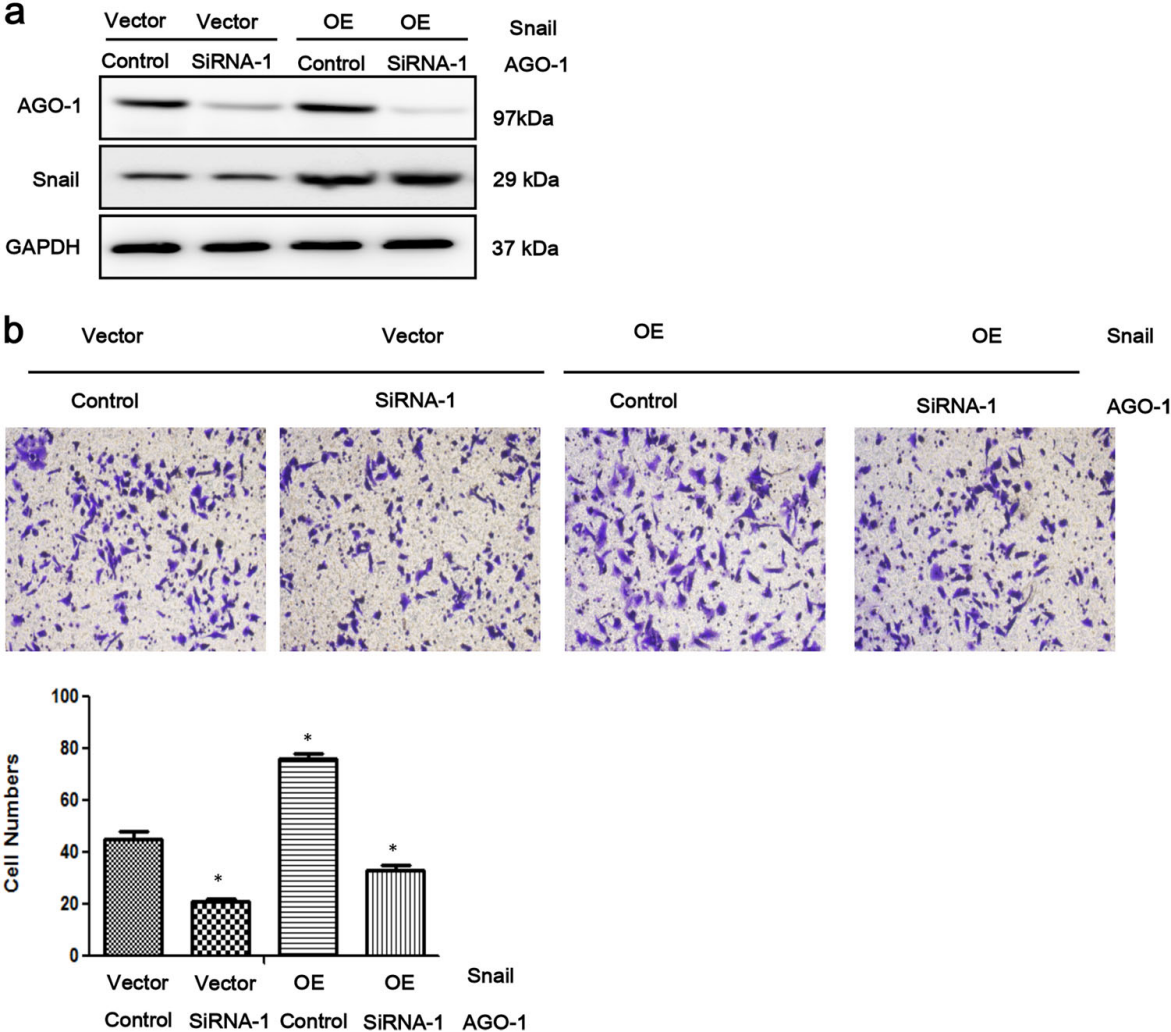
Rescue assay. **a** The expression of Snail after using Snail overexpression and AGO1 siRNAs simultaneously. **b** Snail overexpression significantly promote the cell invasion by transwell assay

After verifying the signal pathway between AGO1 and Snail, we further investigated whether recovering the expression of Snail would rescue the cell invasion, which was inhibited by AGO1 depletion. We stably introduced vector containing cDNA encoding the full-length Snail protein via viral infections into control group and Si-AGO1 cells, respectively, and the resulting cells as mass pools were subjected for in vitro analyses (Fig. 5a). We performed transwell assays to examine the effect of Snail on cell migration. The result demonstrated that Snail overexpression could significantly promote the cell invasion by transwell assay in spite of the downregulation of AGO1 (*P* < 0.001; Fig. 5b).

### IHC findings

**Fig. 6:**
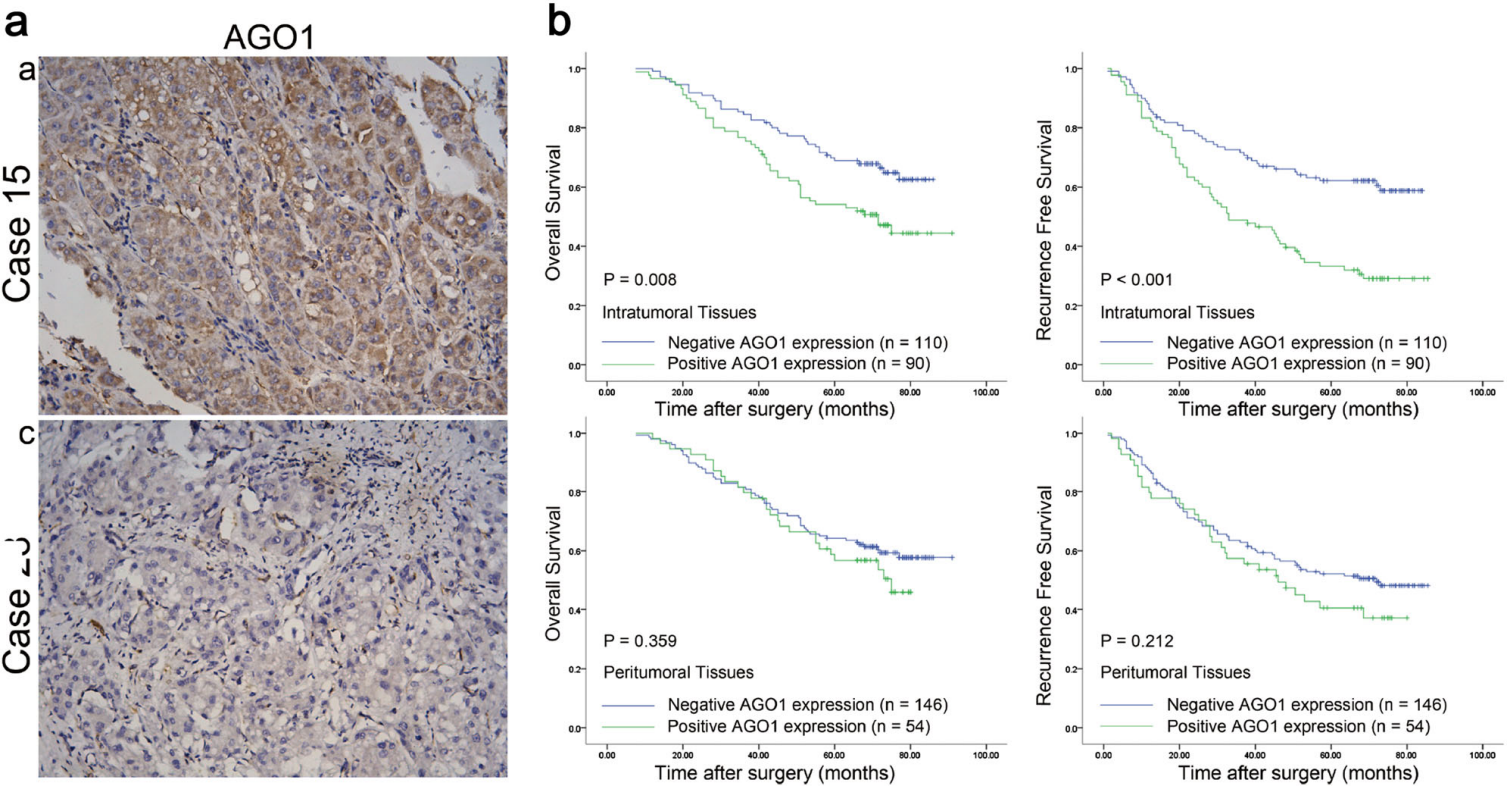
Representative intratumoral immunohistochemical staining of AGO1 and Kaplan–Meier analysis of prognosis. **a** Case 15: an intratumoral tissue with strong positive staining of AGO1; case 28: an intratumoral tissue with negative staining of AGO1. **b** Kaplan–Meier analysis of OS and RFS for AGO1 expression in 200 intratumoral tissue cases. Magnification: ×200

AGO1 staining was most prominent in the cytoplasm of intratumoral HCC cells and adjacent liver cells. Nuclear staining was also detected in several sections. The majority of stromal cells did not stain, but sporadic positive staining was observed (Fig. 6a). The positive rates of AGO1 expression in intratumoral and peritumoral tissues were 45.0% (90 of 200) and 27.0% (54 of 200), respectively. Patients with positive intratumoral AGO1 expression had higher incidences of young age (*P* = 0.039), high serum alpha-fetoprotein (AFP; *P* = 0.002), HBeAg-positive status (*P* = 0.049), HBV DNA ≥ 5 × 10^2^ IU/ml (*P* = 0.043), liver cirrhosis (*P* = 0.045), and intrahepatic or extrahepatic recurrence (*P* < 0.001; Table 2).

**Table 2:** Relationship between AGO1 expression and clinicopathological features

Clinicopathological features	Intratumoral expression of AGO1		
	Negative no.	Positive no.	*P*
Age, years			0.039
≤50	45	50	
>50	65	40	
Gender			0.421
Female	15	16	
Male	95	74	
α-Fetoprotein, ng/ml			0.002
≤20	51	23	
>20	59	67	
HBsAg			0.224
+	84	75	
−	26	15	
HBeAg			0.049
+	22	29	
−	88	61	
HBV DNA, IU/mL			0.043
≥5 × 10^2^	24	32	
<5 × 10^2^	71	49	
HCV			0.820
+	3	2	
−	107	88	
Liver cirrhosis			0.045
+	86	80	
−	24	10	
Tumor size, cm			0.517
≤5	71	62	
>5	39	28	
Tumor number			0.207
Single	90	67	
Multiple	20	23	
Intrahepatic metastasis			0.636
+	12	8	
−	98	82	
Microvascular invasion			0.585
+	21	20	
−	89	70	
Tumor encapsulation			0.755
Complete	38	33	
None	72	57	
Recurrence site			<0.001
No	67	28	
Intrahepatic	37	56	
Extrahepatic	6	6	
Edmondson–Steiner grade			0.387
I–II	81	71	
III–IV	29	19	

*HBsAg* hepatitis B surface antigen, *HBeAg* hepatitis B e antigen, *HCV* hepatitis C virus

### Relationship between AGO1 expression and patient prognosis

The 1-, 3-, 5-, and 7-year overall survival (OS) rates of patients with positive intratumoral AGO1 expression were 97%, 77%, 54%, and 44%, respectively, and were much lower than that in patients with negative intratumoral AGO1 expression (100%, 85%, 70%, and 60%, respectively; *P* = 0.008; Fig. 6b). Furthermore, the 1-, 3-, 5-, and 7-year recurrence-free survival (RFS) rates of patients with positive AGO1 expression (83%, 49%, 34%, and 29%, respectively) were significantly lower than that in patients with negative AGO1 expression (88%, 73%, 62%, and 56%, respectively; *P* < 0.001; Fig. 6b). Multiple tumor number was the risk factor for both OS and RFS in univariate analysis. Positive intratumoral AGO1 expression and tumor number were independent risk factors for both OS and RFS, but peritumoral AGO1 expression was not associated with either OS or RFS (Table 3).

**Table 3:** Univariate and multivariate analyses of factors associated with survival and recurrence

Factor	OS				RFS			
		Multivariate				Multivariate		
	Univariate *P*	Hazard Ratio	95% CI	*P*	Univariate *P*	Hazard Ratio	95% CI	*P*
Age, y (>50 vs ≤50)	0.400			NA	0.319			NA
Gender (female vs male)	0.866			NA	0.886			NA
AFP, ng/ml (>20 vs ≤20)	0.021			NS	0.647			NA
HBsAg (yes vs no)	0.168			NA	0.119			NA
HBeAg (yes vs no)	0.569			NA	0.180			NA
HCV (yes vs no)	0.888			NA	0.618			NA
Liver cirrhosis (yes vs no)	0.043			NS	0.096			NA
Tumor size, cm (>5 vs ≤5)	0.398			NA	0.927			NA
Tumor number (single vs multiple)	<0.001	1.959	1.219–3.147	0.005	<0.001	2.368	1.560–3.594	<0.001
Intrahepatic metastasis (yes vs no)	0.437			NA	0.250			NA
Microvascular invasion (yes vs no)	0.002	1.773	1.094–2.873	0.020	0.082			NA
Tumor encapsulation (complete vs none)	0.289			NA	0.294			NA
Edmondson–Steiner grade (III–IV vs I–II)	0.595			NA	0.837			NA
Intratumoral AGO1 (negative vs positive)	0.008	1.588	1.030–2.450	0.036	<0.001	2.023	1.365–2.999	<0.001
Peritumoral AGO1 (negative vs positive)	0.359			NA	0.212			NA

*NA* not adopted, *NS* not significant

**Fig. 7:**
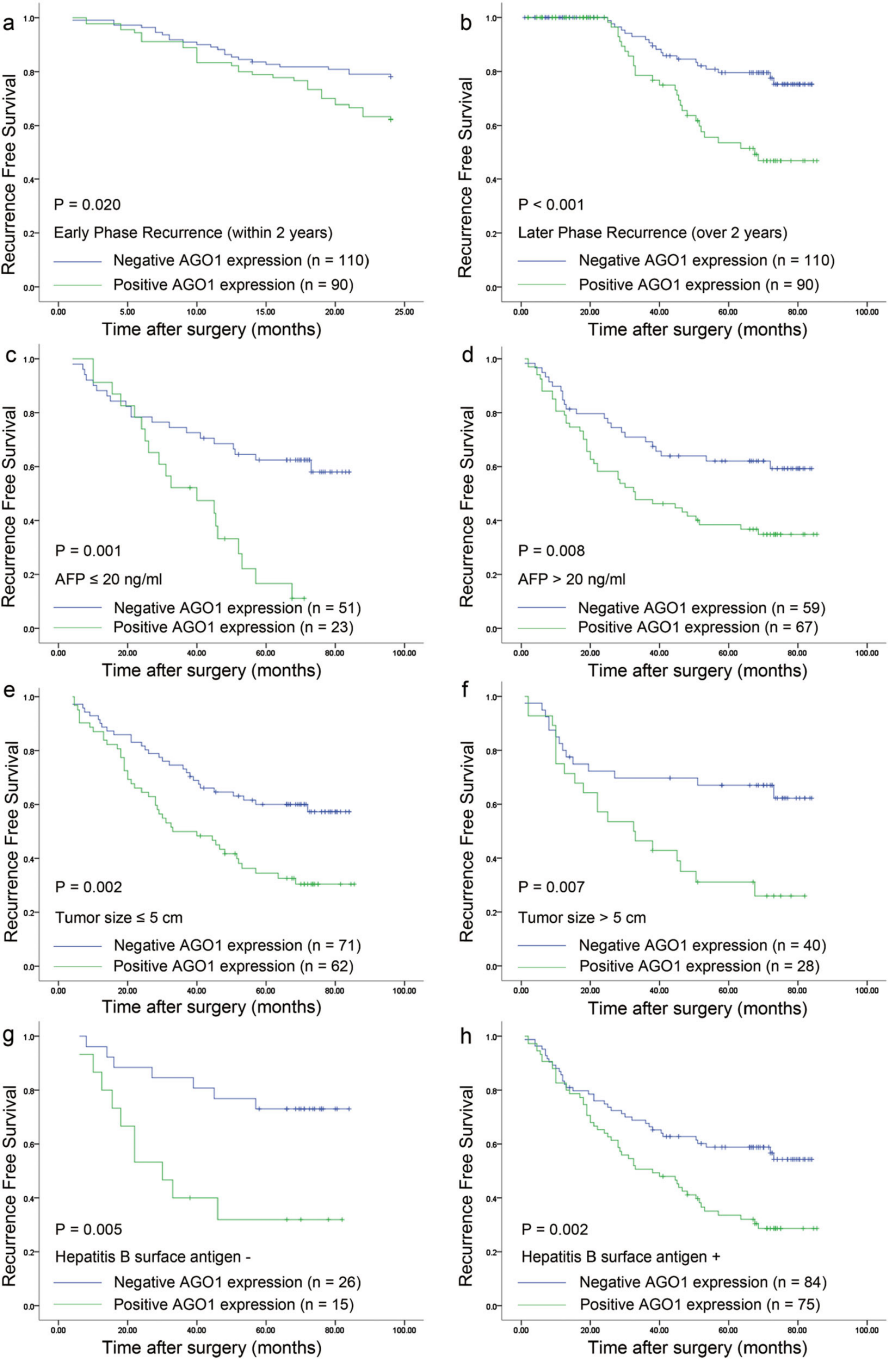
Kaplan–Meier analysis of RFS for AGO1 expression in stratified subgroups. **a**, **b** RFS for AGO1 expression in early- and late-phase recurrence. **c**, **d** RFS for AGO1 expression in the AFP subgroup. **e**, **f** RFS for AGO1 expression in the tumor size subgroup. **g**, **h** RFS for AGO1 expression in the HBsAg subgroup

Twenty-four months was used as a cutoff time point when tumor recurrence was divided into early phase and late phase according to RFS^18^. Positive intratumoral AGO1 expression was associated with a poor early- and late-phase RFS (*P* = 0.020 and *P* < 0.001, respectively; Fig. 7a, b). Furthermore, the predictive value of intratumoral AGO1 expression within subgroups (AFP: ≤20 vs >20 ng/ml; tumor size: ≤5 cm vs >5 cm; HBsAg status: positive vs negative) was investigated, and the prognostic significance of AGO1 was retained: AFP subgroup (*P* = 0.001 and 0.008, respectively; Fig. 7c, d); tumor size subgroup (*P* = 0.002 and 0.007, respectively; Fig. 7e, f); and HBsAg status subgroup (*P* = 0.005 and 0.002, respectively; Fig. 7g, h).

## Discussion

Recently, miRNA-associated genes have attracted attention in HCC research. Dicer is downregulated in HCC intratumoral tissues, and we have shown that Hiwi is associated with HCC metastatic potential and prognosis in patients with HCC^16,19^. Nevertheless, systematic study concerning the role of AGO1 in HCC progression and prognosis has not been reported, specifically with a large number of patients and long-term follow-up.

In the present study, we found that the migration and invasion of HCC cells were dramatically inhibited following suppression of AGO1. Patients with positive intratumoral AGO1 had a higher recurrence rate compared to those with negative intratumoral AGO1. Moreover, AGO1 depletion resulted in significant inhibition of HCC cell proliferation, which is also associated with tumor progression^20^. Our results suggest that upregulation of AGO1 in HCC may correlate with a malignant phenotype and metastasis.

EMT is a major mechanism for carcinogenesis and progression, through which cell proliferation and metastasis, even drug resistance and tumor stem cells generation would be ultimately promoted^21–23^. In our study, we found that AGO1 inhibition would weaken the EMT process, which especially included the crucial transcription factor Snail. Thus, it is necessary to explore the signal pathway that could manipulate the expression of Snail in the cell nucleus. TGF-β pathway is a common signal pathway to manipulating the EMT process, in which includes MAPK proteins and Smad proteins^24,25^. Thus, we further investigated the expression and phosphorylation of TGF-β pathway-associated proteins, including ERK1/2, P38, Smad2, and Smad4, and found that AGO1 could affect the activation of three of them, except for P38. Since Smad2 and Smad4 could play a role in regulating transcription through transfer into the cell nucleus as Smads complex, which one was exactly manipulated by AGO1 should be determined. Then it was confirmed that the quantity of Smad4, but not Smad2, in the cell nucleus and cytoplasm was altered obviously, and through ChIP, we verified that Smad4 could play a role on Snail expression directly combining with its promotor region. The HCC cell functions could be rescued through recovering the expression of Snail. In summary, our results suggest that AGO1 might manipulate EMT process through activating TGF-β pathway-associated proteins, including ERK1/2, Smad2, and Smad4 to promote Smad4 transfer into the cell nucleus and then increase the expression of EMT key transcription factor Snail through combining with its promotor region. Consequently, tumor growth and metastasis of HCC will be dramatically enhanced and the prognosis of HCC patients will be deteriorated.

Among the adverse clinicopathological features positively correlated with AGO1, HBeAg is associated with poor survival in patients with small HCC and a higher risk of early recurrence from intrahepatic metastasis^26^. HBeAg status represents HBV viremia because the presence of HBeAg in serum is associated with active HBV replication in hepatocytes^27^, and this result is further confirmed by the positive relationship between AGO1 expression and the value of HBV DNA in our study. Moreover, a previous study has reported that HBV replication is associated with HCC angiogenesis^28^. Thus, AGO1 may not only promote HCC cell growth and metastasis but also alter the virus-infective status of the HCC environment resulting in increased intrahepatic or extrahepatic metastasis and recurrence.

AGO1 was confirmed to be an independent risk factor for survival in HCC patients, especially for RFS. The high probability of postsurgical recurrence is a major obstacle compromising curative resection for HCC. Recurrence can be categorized as occurring early or late and may occur by different mechanisms, including intrahepatic metastasis and de novo multicentric carcinogenicity. Early postsurgical recurrence occurs within 24 months and is associated with tumor-related factors. Late recurrence occurs after 24 months and is associated with hepatic inflammation and HBV replication^29^. We propose that the association between AGO1 and intrahepatic metastasis and HBV replication suggests that AGO1 may be a reliable prognostic tool regardless of recurrence time. Early postsurgical recurrence is the leading cause of death from HCC, including small HCC^30^, and our results may highlight a novel marker that will identify a high-risk subgroup of patients who require adjuvant therapies following curative resection.

Similar to early recurrence, a reliable marker is also needed to predict prognosis in subgroups of normal AFP and small HCC. AFP is a common tumor marker for monitoring postsurgical recurrence and metastasis in patients with positive AFP^31^. However, there is not an ideal prognostic marker for patients with normal serum AFP (30–40% of all), making them difficult to screen for recurrence or prognosis^32–34^. Similar to patients with normal AFP, the recurrence rate of small HCC after curative resection is still high, and a number of studies are searching for suitable markers to predict postsurgical recurrence^35,36^. The majority of HCC patients in China have HBV-positive backgrounds, unlike patients from other countries^1^. We stratified our patients according to HBsAg status, and the prognostic role of AGO1 was validated in HCC patients with an HBV-negative background. These results indicate that AGO1 may be an applicable and reliable prognostic biomarker of HCC regardless of serum AFP levels, tumor size, early or late postsurgical recurrence, or HBV background.

Although we found the adverse effect of AGO1 in this study, there are several previous studies that have some opposite conclusions. Chen et al.^37,38,]^ found that AGO1 may inhibit the expression and function of vascular endothelial growth factor in human umbilical vein endothelial cells (HUVEC), and AGO1 was a marker for better OS and disease-free survival in HCC. Another study found overexpression of AGO1 could reduce tumor growth in animal models. We considered that the difference of research methods (down- and upregulation), cells (HCC cell lines and HUVEC), and patient samples’ basic characters may cause the difference between our study and these studies. Furthormore, a recent study also found AGO1 expressing in the cell nucleus could interact with RNA polymerase to regulate gene expression, which was totally different from AGO1 in the cytoplasm. Inhibition of AGO1 could reduce cancer cell progress and survival^39^. Therefore, dual role of AGO1 in cancers could not be excluded and further study is necessary.

In conclusion, our data show the potential of AGO1 to be a predictive biomarker in HCC patients with long-term follow-up. Our data suggest that AGO1 may play a crucial role in regulating HCC cell growth and metastasis through TGF-β pathways. AGO1 may be a reliable prognostic factor for HCC.

## Electronic supplementary material

supplemental figure 1

supplemental figure 2
