# Supplementary figures and images for: AGO1 may influence the prognosis of hepatocellular carcinoma through TGF-β pathway
Source: Cell Death Dis. 2018 Feb 27;9(3):324. doi: 10.1038/s41419-018-0338-y (PMC5832432; doi:10.1038/s41419-018-0338-y)

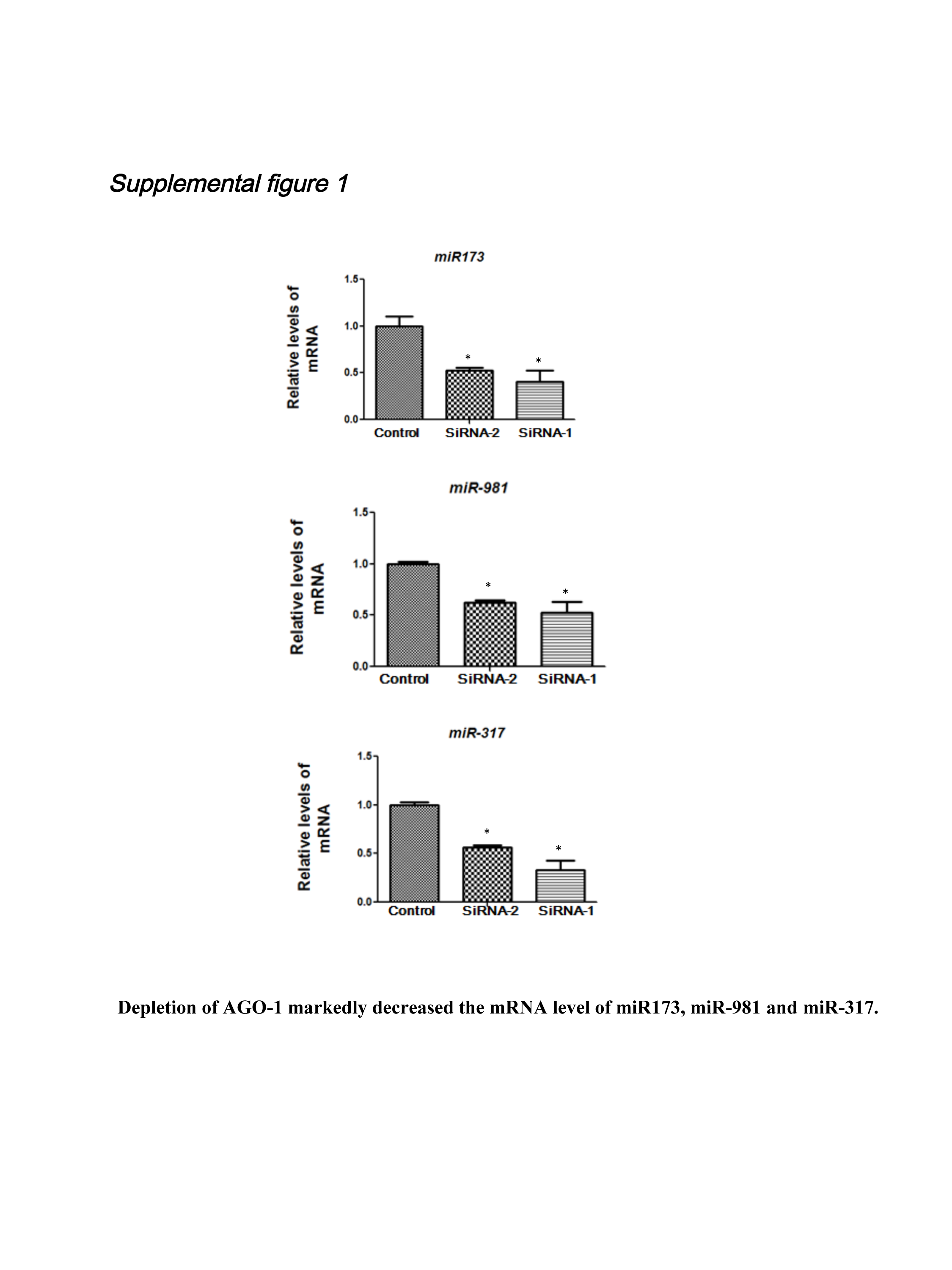

Supplement: Supplementary file 1 — supplemental figure 1 [file 41419_2018_338_MOESM1_ESM.tif]

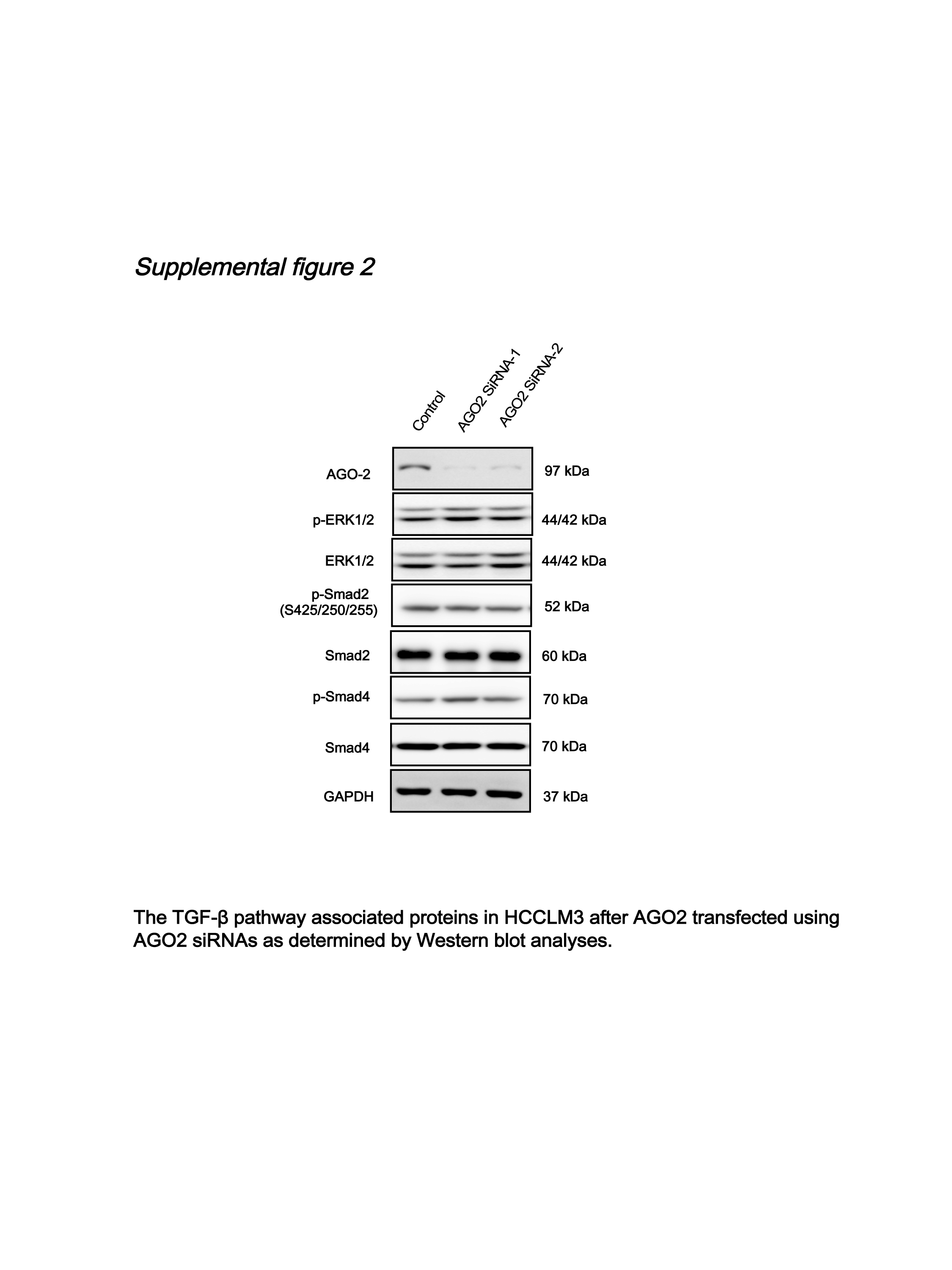

Supplement: Supplementary file 2 — supplemental figure 2 [file 41419_2018_338_MOESM2_ESM.tif]
